# Vici syndrome in Israel: Clinical and molecular insights

**DOI:** 10.3389/fgene.2022.991721

**Published:** 2022-09-20

**Authors:** Odelia Chorin, Yoel Hirsch, Rachel Rock, Liat Salzer Sheelo, Yael Goldberg, Hanna Mandel, Tova Hershkovitz, Nicole Fleischer, Lior Greenbaum, Uriel Katz, Ortal Barel, Nasrin Hamed, Bruria Ben-Zeev, Shoshana Greenberger, Nadra Nasser Samra, Michal Stern Zimmer, Annick Raas-Rothschild, Ben Pode-Shakked

**Affiliations:** ^1^ The Institute for Rare Diseases, Edmond and Lily Safra Children’s Hospital, Sheba Medical Center, Ramat Gan, Israel; ^2^ The Danek Gertner Institute of Human Genetics, Sheba Medical Center, Ramat Gan, Israel; ^3^ Sackler Faculty of Medicine, Tel-Aviv University, Tel Aviv-Yafo, Israel; ^4^ Dor Yeshorim, Committee for Prevention of Jewish Genetic Diseases, New York, NY, United States; ^5^ Raphael Recanati Genetic Institute, Rabin Medical Center—Beilinson Hospital, Petah Tikva, Israel; ^6^ Unit of Inherited Metabolic Disorders, Ziv Medical Center, Safed, Israel; ^7^ Institute of Human Genetics, Ziv Medical Center, Safed, Israel; ^8^ The Genetics Institute, Rambam Health Care Campus, Haifa, Israel; ^9^ Ruth and Bruce Rappaport Faculty of Medicine, Technion Institute of Technology, Haifa, Israel; ^10^ FDNA Inc, Boston, MA, United States; ^11^ The Joseph Sagol Neusroscience Center, Sheba Medical Center, Ramat Gan, Israel; ^12^ Pediatric Heart Institute, Edmond and Lily Safra Children’s Hospital, Sheba Medical Center, Ramat Gan, Israel; ^13^ The Genomic Unit, Sheba Cancer Research Center, Sheba Medical Center, Ramat Gan, Israel; ^14^ The Wohl Institute for Translational Medicine and Cancer Research Center, Sheba Medical Center, Ramat Gan, Israel; ^15^ Pediatric Neurology Unit, Edmond and Lily Safra Children’s Hospital, Sheba Medical Center, Ramat Gan, Israel; ^16^ The Talpiot Medical Leadership Program, Sheba Medical Center, Ramat Gan, Israel; ^17^ Department of Dermatology, Sheba Medical Center, Ramat Gan, Israel; ^18^ Azrieli Faculty of Medicine, Bar-Ilan University, Safed, Israel; ^19^ Pediatric Department B, Edmond and Lily Safra Children’s Hospital, Sheba Medical Center, Ramat Gan, Israel

**Keywords:** EPG5, Vici syndrome, congenital cataract, agenesis of corpus callosum, global developmental delay, cardiomyopathy, autophagy, recurrent Ashkenazi Jewish mutation

## Abstract

**Introduction:** Vici Syndrome is a rare, severe, neurodevelopmental/neurodegenerative disorder with multi-systemic manifestations presenting in infancy. It is mainly characterized by global developmental delay, seizures, agenesis of the corpus callosum, hair and skin hypopigmentation, bilateral cataract, and varying degrees of immunodeficiency, among other features. Vici Syndrome is caused by biallelic pathogenic variants in *EPG5*, resulting in impaired autophagy. Thus far, the condition has been reported in less than a hundred individuals.

**Objective and Methods:** We aimed to characterize the clinical and molecular findings in individuals harboring biallelic *EPG5* variants, recruited from four medical centers in Israel. Furthermore, we aimed to utilize a machine learning-based tool to assess facial features of Vici syndrome.

**Results:** Eleven cases of Vici Syndrome from five unrelated families, one of which was diagnosed prenatally with subsequent termination of pregnancy, were recruited. A total of five disease causing variants were detected in *EPG5*: two novel: c.2554-5A>G and c.1461delC; and 3 previously reported: c.3447G>A, c.5993C>G, and c.1007A>G, the latter previously identified in several patients of Ashkenazi-Jewish (AJ) descent. Amongst 140,491 individuals screened by the Dor Yeshorim Program, we show that the c.1007A>G variant has an overall carrier frequency of 0.45% (1 in 224) among AJ individuals. Finally, based on two-dimensional facial photographs of individuals with Vici syndrome (*n* = 19), a composite facial mask was created using the DeepGestalt algorithm, illustrating facial features typical of this disorder.

**Conclusion:** We report on ten children and one fetus from five unrelated families, affected with Vici syndrome, and describe prenatal and postnatal characteristics. Our findings contribute to the current knowledge regarding the molecular basis and phenotypic features of this rare syndrome. Additionally, the deep learning-based facial gestalt adds to the clinician’s diagnostic toolbox and may aid in facilitating identification of affected individuals.

## Introduction

Vici Syndrome (OMIM #242840) is a severe, rare neurodevelopmental/neurodegenerative disorder with multi-systemic manifestations presenting in infancy, and characterized by profound global developmental delay (GDD), agenesis of the corpus callosum (ACC), hair and skin hypopigmentation and bilateral cataracts (Dionisi-Vici et al., 1988). Additional features include progressive microcephaly, failure to thrive, cardiomyopathy and varying degrees of immunodeficiency (del Campo et al., 1999; Chiyonobu et al., 2002; Byrne et al., 2016a; Abidi et al., 2020). The condition was first described by the Italian physician Carlo Dionisi-Vici and colleagues in 1988, in two Italian brothers who died at 2 and 3 years of age due to bronchopneumonia. Since then, less than a hundred cases have been described worldwide, with a global prevalence of less than 1/1,000,000 (Alzahrani et al., 2018).

The disorder is caused by biallelic pathogenic variants in the *EPG5* gene, located on 18q12.3-q21.1, which encodes for ectopic P granules autophagy protein 5. This protein plays a role in autophagosome-lysosome fusion, and impaired function of this protein disables degradation within the lysosomes (Tian et al., 2010; Cullup et al., 2013). Impaired autophagy may have a range of clinical consequences including impairment of the innate immune response, aggregation of misfolded or other harmful proteins leading to degenerative processes, and impaired response to stress, leading to tissue toxicity (Mizushima, 2007).

We present herein 11 cases from five unrelated families affected by Vici Syndrome, one of which was diagnosed prenatally with subsequent termination of pregnancy. Along with their molecular and clinical findings, we show a high carrier rate of the c.1007A>G variant in the Ashkenazi-Jewish (AJ) population, and provide a facial composite mask specific to patients with Vici syndrome, based on deep learning technology.

## Patients and methods

### Patient recruitment

Patients with Vici Syndrome were recruited through collaborative efforts of four medical centers in Israel (Sheba Medical Center, Rabin Medical Center, Rambam Health Care Campus, Ziv Medical Center), that have identified patients with Vici Syndrome. Inclusion criteria included patients with clinical findings consistent with Vici Syndrome confirmed by molecular testing (identified biallelic pathogenic variants in *EPG5* in the patient or in one case, family history of pathogenic biallelic *EPG5* mutations). Clinical data was obtained from questionnaires filled out by participating physicians and based on physical examination, medical history and imaging data. Clinical facial photographs and imaging studies are presented following parental informed consent for their publication.

The study was performed under the ethical guidelines of the participating medical centers for the conduction of clinical studies.

### Molecular diagnosis

Following parental informed consent, blood samples (9/11 of cases) or fetal amniocytes (1/11 cases) were obtained, and DNA was extracted according to standard procedures. Targeted sequencing of the *EPG5* gene was conducted for patients A1, A2, B2, E1, E2, E3, E4, and E5 by Sanger Sequencing according to standard procedures. Exome Sequencing (ES) was performed for patients C1, D1 on genomic DNA at the Bioinformatics Unit, Sheba Medical Center, using the Twist Human Core Exome Plus Kit (Twist Bioscience, San Francisco, CA, United States), as previously described (Pode-Shakked et al., 2021).

### Prevalence of c.1007A>G, p.Gln336Arg variant in the Ashkenazi-Jewish population

In order to evaluate the carrier rate of the c.1007A>G, p.Gln336Arg variant in the AJ population, we reanalyzed Next Generation Sequencing (NGS)-based testing data of 140,491 individuals from diverse Jewish populations who enrolled in the Dor Yeshorim program between 2016 and 2021. The Dor Yeshorim program aims to test individuals of different Jewish subgroups pre-marriage in order to avoid mating of disease-causing variant carriers of the same gene, thereby decreasing risk for recessively inherited disorders.

### Generation of facial gestalt of Vici syndrome using machine learning

Utilizing the research application of the DeepGestalt algorithm used in the Face2Gene platform (FDNA Inc., MA, United States), a facial gestalt typical to patients with Vici syndrome was generated. For this purpose, we uploaded twenty 2-dimensional (2D) frontal facial photos of Vici syndrome patients, either from the cohort reported herein or those previously published (*n* = 19), in a de-identified manner. Then, the proprietary DeepGestalt (v.19.1.3) algorithm was used to create a descriptor (mathematical representation) of the face, as previously described for other genetic disorders (Gurovich et al., 2019).

## Results

**FIGURE 1:**
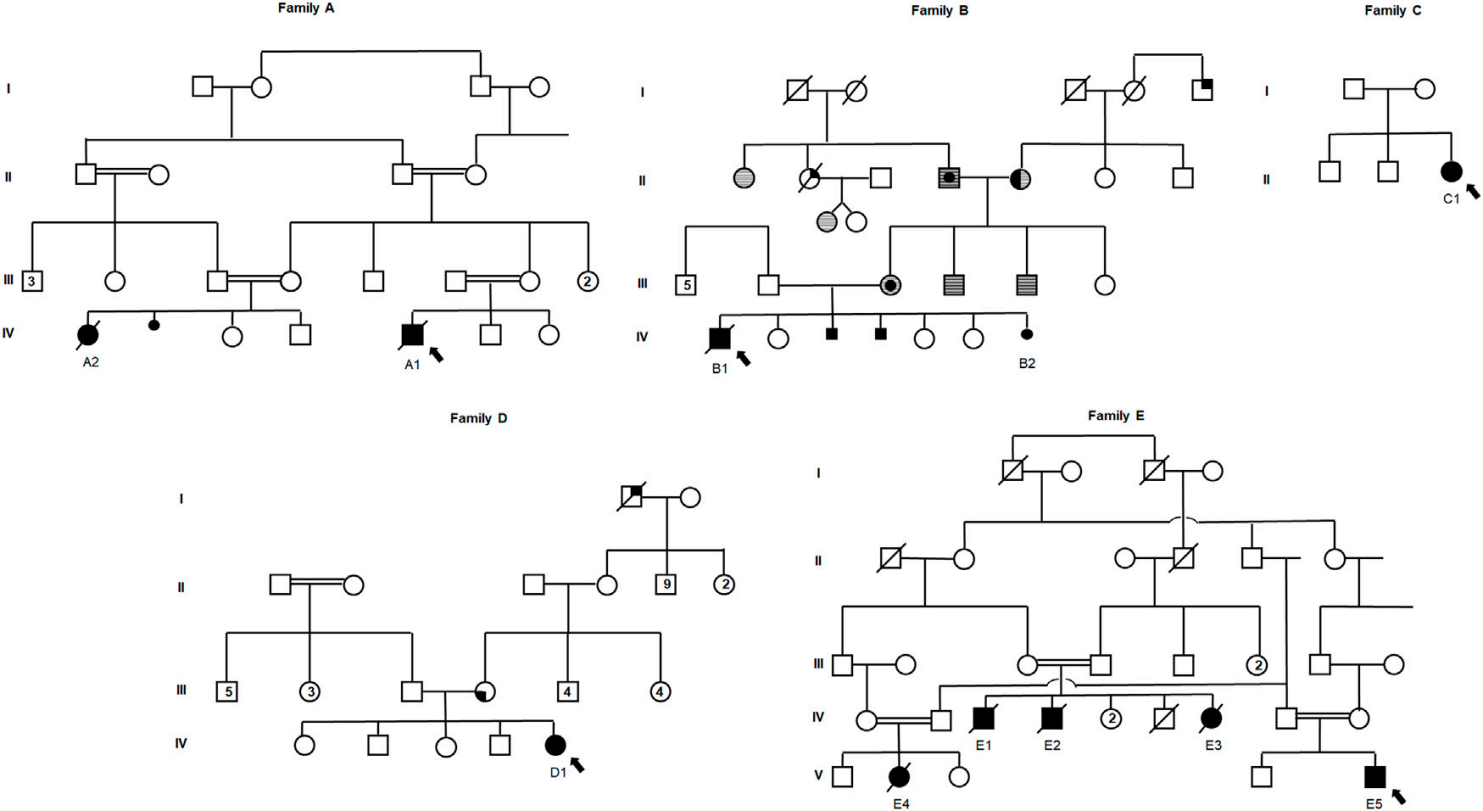
Pedigrees of five unrelated families with a molecular diagnosis of Vici syndrome. Full symbols designate individuals affected with Vici syndrome. Symbols with a central circle designate individuals with melanoma; symbols with full right upper quadrant designate individuals with colorectal cancer; symbols with full left lower quadrant designate individuals with hepatic tumor; horizontally-striped symbols designate individuals with gastrointestinal polyps; half-full symbols designate individuals with thyroid cancer. Smaller full circle or square symbols designate fetuses who were unborn or pregnancies that did not complete term, either female or male, respectively. Double-lines depict consanguinity. Arrows depict the proband of each family.

This series includes 11 cases from five unrelated families presenting with Vici Syndrome, one of which was diagnosed prenatally. Family pedigrees are depicted in Figure 1. Detailed clinical and molecular characteristics of the eleven cases are presented in Supplemental Table S1 and Supplemental Materials.

### Clinical characteristics

In Family A, probands A1 and A2 are first degree cousins born to consanguineous parents of Arab-Muslim descent. The parents in Family B (Cases B1 and B2) are a non-consanguineous couple of AJ descent. Of these, B2 was diagnosed prenatally and following a genetic molecular diagnosis, termination of pregnancy (TOP) was performed. Notably, two additional pregnancies of the couple (family B) that were terminated following prenatal findings of partial ACC. Unfortunately, further data regarding their prenatal features or molecular diagnosis were unavailable, although family history is highly suggestive of recurring Vici syndrome in these two fetuses. In Families C and D, the probands (C1 and D1, respectively) are singleton female patients born to AJ parents. Finally, Family E is a highly consanguineous extended family of Arab-Muslim descent, of which cases E1, E2, and E3 are siblings, while Cases E4 and E5 are their first degree cousins (Figure 1). Of these 11 cases, A1, A2, and E1-E4 were previously reported by Byrne et al., 2016b.

Our case series includes five females, a female fetus, and five males. Physical features included hypopigmentation amongst 9/10 postnatal cases, cataract in 9/10 postnatal cases and was also evident in the aborted fetus (B2), along with additional recurring dysmorphic facial features. Hearing loss was reported amongst four cases).

Prenatal findings included ACC (B2, C1, and E5) and dysgenesis of the brainstem and cerebellum, dilated third ventricle, and colpocephaly of cerebral ventricles in case C1. In both cases C1 and E5, parents received genetic counseling, with a high likelihood for Vici Syndrome in case E5, and opted to proceed with the pregnancy without additional testing.

All 10 postnatal cases presented with profound global developmental delay. Seizures were reported amongst all cases for which data was available (A1, A2, B1, D1, and E5); in one case with resistance to multiple treatment regimens (D1). Myopathy, evident by elevated CPK levels, was documented in 8/10 cases in which data was available, along with variable additional laboratory abnormalities. Brain imaging revealed ACC in all reported cases, with additional brain abnormalities in five cases: pontine cerebellar hypoplasia and ventriculomegaly (A1), large citerna magna and delayed myelination (A2), dysgenesis of brainstem and cerebellum, dilated third ventricle and colpocephaly of cerebral ventricles (C1), enlarged lateral ventricles, brain atrophy and agenesis of septum pellucidum (D1).

Cardiomyopathy was evident in all but one postnatal case, with 7/9 cases presenting with variable degrees of hypertrophic cardiomyopathy, and two cases presenting with dilated cardiomyopathy. Postnatal growth retardation and microcephaly were documented in all cases where data was available (A1, A2, C1, and D1) and all 10 postnatal cases underwent PEG insertion. Immune deficiency was shared by all postnatal cases, involving recurrent respiratory and gastrointestinal infections among others.

Families B and D reported of several cases of malignancies, including recurrent melanoma in the mother and maternal grandfather, colorectal cancer in a sibling of the maternal grandfather, papillary thyroid carcinoma in the maternal grandmother and multiple persons with colorectal polyps (mother, maternal uncles, maternal grandparents) in family B; maternal invasive liver cancer (age 29) and a maternal grandfather diagnosed with lung and colon cancer at the age of ∼75 years in family D.

### Molecular diagnosis

For Family A, targeted Sanger sequencing of *EPG5* (NM_020964.3, Hg38) was conducted for both patients and revealed the following homozygous pathogenic variant: Chr18-45916144C>T, c.3447G>A, p.Trp1149*. This variant has been previously reported by Byrne et al. (2016a) within this family and classified as pathogenic in ClinVar (VCV000812308.1).

In Family B, diagnosis was carried out for the aborted fetus (B2), found to be compound heterozygous for the following *EPG5* variants: Chr18-45954395T>C, c.1007A>G, p.Gln336Arg, found to be paternally inherited; and a maternally-inherited Chr18-45925907T>C, c.2554-5A>G. The c.1007A>G variant has been reported previously in various publications (Byrne et al., 2016a; Byrne et al., 2016b), and suggested to be a founder Ashkenazi mutation, and was classified as likely pathogenic/pathogenic (RCV000702544). The c.2554-5A>G variant is rare in population databases (one heterozygous carrier in gnomAD) (PM2) and is expected to alter splicing in multiple bioinformatic tools (PP3), including SpliceAI (acceptor loss 0.35-5bp, acceptor gain 0.99-1bp) and VarSEAK (class 5), with a TraP score of 0.478 and CADD score of 24. It is in trans with an established pathogenic variant (PM3) and patient’s phenotype and family history are highly specific for this disease (PP4). Based on these criteria, this variant was classified as likely pathogenic.

For Family C, trio ES was pursued and the proband was found to harbor the following homozygous variant in *EPG5*: Chr18-45954395T>C, c.1007A>G, p.Gln336Arg, similar to the maternally-inherited variant in Family B.

For Family D, following clinical suspicion of Vici syndrome and in light of the AJ origin, the proband was initially tested for the aforementioned c.1007A>G variant in *EPG5* and found negative. Then, single (patient-only) ES was pursued, revealing the proband to harbor a homozygous variant in *EPG5*: Chr18-45949519 CG>C, c.1461delC, p.Ala488LeufsTer32. This variant is absent from population databases of healthy individuals (PM2) and is expected to cause premature termination of the protein, leading to nonsense mediated decay (PVS1). The patient’s phenotype is highly specific for this disease (PP4). Based on these criteria, this variant is classified as likely-pathogenic.

For Family E, targeted Sanger sequencing of *EPG5* (NM_020964.3, Hg38) was conducted for the affected individuals and revealed the following homozygous pathogenic variant: Chr18-45876292G>C, c.5993C>G, p.Ser 1998*. This variant has been previously reported by Byrne et al. (2016b) within this family and classified as likely pathogenic in ClinVar (RCV000768386).

### Ashkenazi-Jewish mutation

Amongst the 140,491 individuals within the Dor Yeshorim program that were tested for the c.1007A>G, p.Gln336Arg mutation, 508 carriers were identified, yielding a carrier frequency of 0.36% (1 in 276). When further refining ancestry groups, the carrier rate is 0.45% (1 in 224) AJ individuals, 0.22 (1 in 454) in mixed Ashkenazi/Sephardi Jewish individuals and 0% in Sephardi Jewish individuals. Within the Ashkenazi population, there was a high variation in number of samples per country of origin (44 samples from individuals of Belarus origin and 8,930 samples from individuals of Hungarian origin). The highest carrier frequency 1.02% (1 in 98) was noted amongst individuals from Polish origin, reaching significance when compared to Hungarian carrier frequency (Fishers’s exact test *p* = 1).

### Generation of facial gestalt of Vici syndrome using machine learning

A composite descriptor (mathematical representation) of the face typical to Vici syndrome was created using the DeepGestalt algorithm (Figure 1C), demonstrating some of the unique facial features, including pale skin and bright hair, anteverted nostrils and long philtrum. Additionally, using the research platform of Face2Gene, the facial gestalt was compared to a sex- and age-related control cohort of 19 unaffected children. As facial gestalt has been shown to be affected by ethnic background (Lumaka et al., 2017), the control cohort was also ethnically-matched. The two cohorts achieved an almost perfect separation, with a *p*-value of <0.0001 (Supplemental Figure S1). This means that the technology views the cohorts as two totally separate groups in terms of the facial phenotype.

## Discussion

We present herein eleven cases (ten affected individuals and a fetus) with a clinical and molecular diagnosis of Vici syndrome. While approximately 80 patients have thus far been reported in the literature, our experience provides several insights into this unique and rare disorder.

Families A, D and E presented with a homozygous null variant. Patients with biallelic loss of function mutations have been reported to develop severe cardiomyopathy and immune deficiency, with a significantly reduced life expectancy (Byrne et al., 2016a; Vojcek et al., 2020). In this regard, it is worthy to note that Patient D1 presented as early as 6 weeks of age with rapid progression of cardiomyopathy requiring multiple medications (Atenolol, Disopyramide and Amlodipine).

Family C presented with the homozygous c.1007A>G variant, previously reported and suggested to be an AJ founder mutation, and the most common recurring mutation causing Vici syndrome (Byrne et al., 2016a; Byrne et al., 2016b). This mutation causes an amino acid substitution (p.Gln336Arg) which is thought to cause alternative splicing, maintaining a normally spliced product in 25% of the transcribed RNA, leading to a full length EPG5 protein, while 75% of the mRNA undergoes nonsense mediated decay due to aberrant spliced isoforms (Byrne et al., 2016b; Kane et al., 2016). Furthermore, it has been shown that this substitution maintains the overall structure, thermal stability and binding capacities of the proteins to GABARAP-y (Nam et al., 2021). The remaining functioning isoform may explain the previously reported milder phenotype with prolonged survival (Byrne et al., 2016a; Vojcek et al., 2020). In accordance with prior reports, this patient developed mild dilated cardiomyopathy, and currently receives no treatment and visual involvement developed at ∼12 months of age.

The recurring c.1007A>G (p.Gln336Arg) variant in *EPG5* appears in gnomAD (v2.11) with a maximal allele frequency of 0.001584 (1 in 631) amongst persons of AJ descent (13 of 8,206 persons within the cohort). In the Dor Yeshorim dataset, which includes 140,491 individuals of Jewish descent, the Ashkenazi population frequency is ∼0.0045 (1 in 224) of this variant, with a maximal subpopulation frequency of 0.01 (1 in 98) amongst individuals from Polish origin. This variant is also present in a high rate amongst non-Finnish European subpopulations, at a frequency of 0.0001055 (3 in 28,434). The higher incidence amongst Ashkenazi Polish descendants in comparison to other Ashkenazi subpopulations (e.g., Hungarian) also supports this variant as a founder mutation within this subgroup.

Considering the recessive inheritance of Vici syndrome, and the carrier frequency of the recurring c.1007A>G (p.Gln336Arg) variant, the expected incidence rate would be 1/200,704. However, less than 80 cases have been reported thus far. The reasons for this discrepancy are not fully understood. The decreased birth incidence raises the possibility of prenatal demise of fetuses carrying biallelic pathogenic variants, perhaps due to multiple malformations and induced or spontaneous abortions. A similar phenomenon has been described for Smith-Lemli-Opitz syndrome (SLOS, OMIM #270400), with a carrier frequency of 0.023 (1 in 43) amongst Ashkenazi Jews, with an expected incidence rate of 1/7,396 (Lazarin et al., 2017). However, published incidence rates range from 1/20,000 to 1/101,000 (Opitz et al., 1987; Nowaczyk et al., 2001) and prenatal demise is suspected amongst 42%–88% of affected conceptuses. An additional potential contributor to the observed incidence rate being less than expected might be underdiagnosis of this disorder.

Consistent with our findings (cases B2, C1, E5) prenatal diagnosis of ACC has been previously reported in cases of Vici syndrome. We broaden the clinical spectrum of fetal presentation of Vici Syndrome to include dysgenesis of the brainstem and cerebellum, a dilated third ventricle and colpocephaly of the cerebral ventricles (C1). These findings partially overlap with those of a prenatal report by Touraine et al. (2017) of a fetus carrying a homozygous c.5870-1G>A variant in *EPG5*. Fetal MRI was performed at 29 weeks of gestation to their reported fetus, revealing ACC, lack of gyration development with abnormal Sylvian fissures and enlarged pericerebral spaces, as well as pontocerebellar hypoplasia involving mainly the brainstem. Additional findings reported during their autopsy included nascent cataract, in accordance with the fetal findings of Case B2. Taken together, these findings underscore that the clinical spectrum of Vici syndrome in its fetal presentation may include structural brain anomalies beyond callosal agenesis.

**FIGURE 2:**
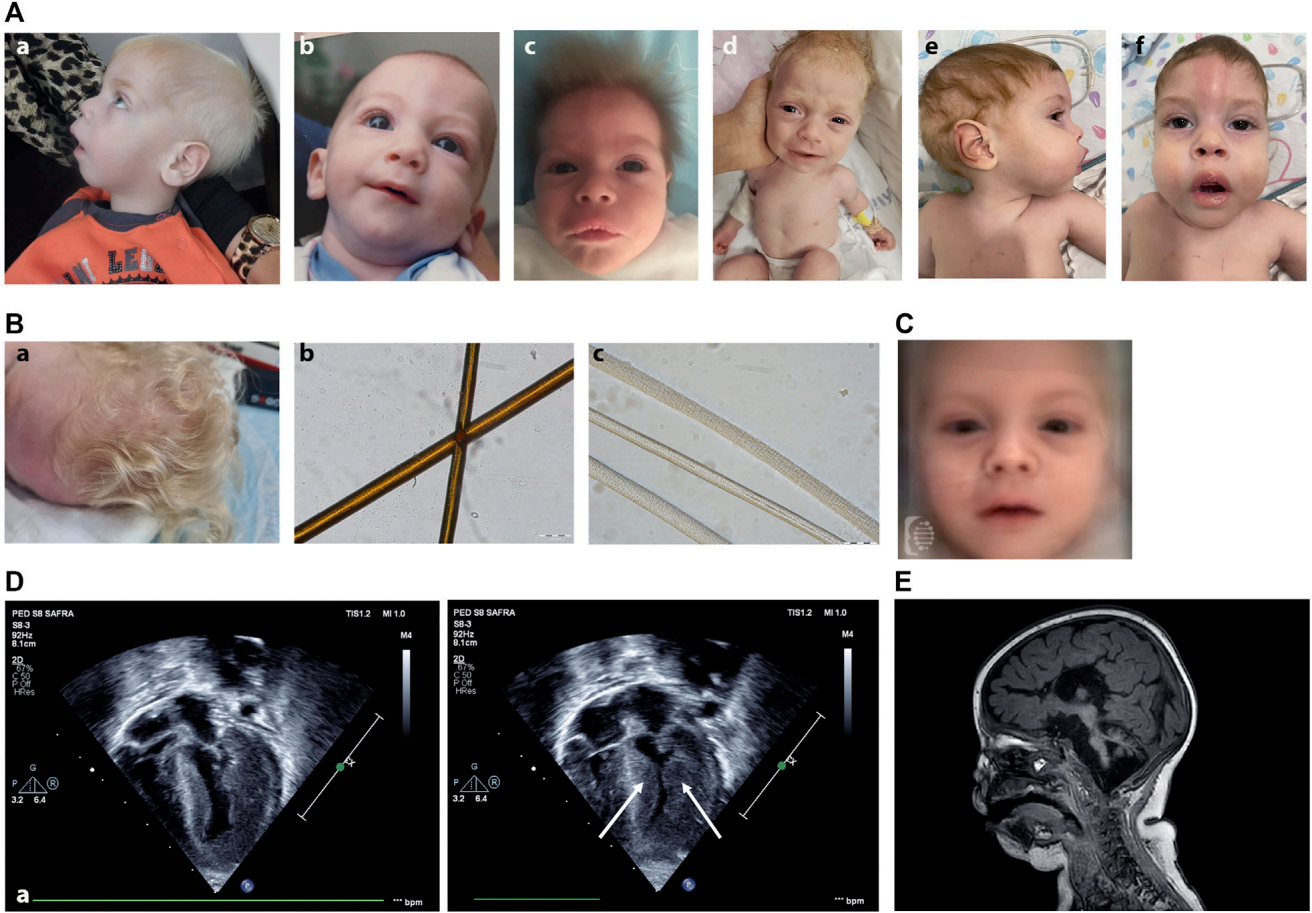
**(A)** Clinical photographs of patients with Vici syndrome. (a) Patient A2. (b) Patient B1 at the age of 12 months. (c) Patient C1 at the age of 1.5 months. (d) Patient D1 at the age of 6 weeks. (e,f) Patient E5 at the age of 15 months. Note the small nose, anteverted nares, pale skin, and bright, upright-positioned hair. **(B)** Hair morphology and hair microscopy in Vici syndrome. (a) Clinical picture of the hair of patient D1 showing hypopigmented hair in normal texture. (b,c) Hair shafts light microscopy (200x). Hair sample of normal control hair, showing evenly distributed pigment (b) and hair shafts of patient D1 showing paucity of pigment (c) (Scale bar: 100 μm). **(C)** Facial composite mask of Vici syndrome created using the DeepGestalt algorithm, based on frontal photos of 19 patients. Note the pale skin and bright hair, anteverted nostrils and long philtrum. **(D)** 4D echocardiogram of patient D1 demonstrating severe left ventricular hypertrophy (arrow). **(E)** Sagittal Brain MRI image (T1) of Patient D1 (8 weeks old) displaying agenesis of the corpus callosum, pontocerebellar hypoplasia, absence of septum pellucidum, brain atrophy and delayed myelination.

While the facial appearance of patients with Vici syndrome may vary, several mild dysmorphic features have been noted to be shared by some previously reported patients, including a long philtrum, anteverted nares, full lips and macroglossia (Byrne et al., 2016a). Together with the general hypopigmentation of the hair and skin, displaying paucity of pigment by light microscopy (Figure 2B), it is our experience that some children with Vici syndrome may be strikingly similar to one another, occasionally making the syndromic appearance recognizable. In order to validate this observation using next-generation phenotyping technology implementing deep learning, we utilized the proprietary DeepGestalt algorithm of the Face2Gene platform. This yielded, for the first time, a machine-learning-based composite mask (“gestalt”) typical to patients with Vici syndrome (Figure 2C). This not only demonstrates the recognizable features of this unique disorder, but contributes to the application of automated technology such as FDNA in identifying future patients, and further adds to the clinician’s toolbox as it may serve to raise or support a clinical suspicion of this diagnosis.

An additional notable finding broadening the known phenotype of Vici syndrome is that of high arched palate, noted in 4 of the affected individuals in this cohort. While the two original siblings reported by Dionisi-Vici had cleat lip and palate (Dionisi-Vici et al., 1988), palatal abnormalities were not noted in the vast majority of patients reported in the literature to date. Our finding of a high arched palate may be part of this spectrum.

A few additional clinical features not yet considered part of the Vici syndrome phenotype were each noted in a single patient within our cohort. These include skeletal findings, such as congenital developmental dislocation of this hip (DDH) (in Patient A1), arachnodactyly (Patient D1), widespread contractures (Patient C1) and polydactyly (Patient A2). The latter is noteworthy as syndactyly has previously been reported in two families with Vici Syndrome (Cullup et al., 2013; Byrne et al., 2016a). Nonetheless, as each of these was only noted in a single patient in our cohort, and given the rarity of the syndrome, no definitive conclusions can be drawn as to whether these constitute rare manifestations of Vici syndrome or are alternatively coincidental.

Of note, in two of the families presented herein (families B and D), family history is significant for multiple cases/early onset cancer (amongst carriers). This finding is in line with a preliminary report by Byrne and colleagues (2016) of a possible association to increased cancer risk amongst carriers of heterozygous *EPG5* pathogenic mutations. The protein encoded by *EPG5* plays a key role in autophagy and is thought to act as a tethering factor which enables the subsequent fusion between autophagosomes and lysosomes (Wang et al., 2016; Nam et al., 2021). Autophagy has been implicated in suppression of cancer initiation, by preventing toxic accumulation within multiple organelles, in particular the mitochondria (Klionsky et al., 2021; Mizushima et al., 2021). Indeed, this gene was originally reported with an association to breast cancer (Halama et al., 2007). Interestingly, increased cancer risk has not been reported in the recurrent Ashkenazi mutation, c.1007A>G, p.Gln336Arg. While data is scarce, this may be secondary to the residual function of the *EPG5* protein (Byrne et al., 2016b; Kane et al., 2016). Further studies are needed to better understand this mechanism and risk assessment, in order to provide counseling and provide recommendations regarding the required surveillance regimens.

The patients reported here present with wide phenotypic variability, which includes the absence of the core features of hypopigmentation in one family (family B), absence of cataract in one case (A2) (family A), intrafamilial variability (family E) as well as variable dysmorphic features and congenital malformations. Based on the above data, we cannot draw conclusions regarding genotype-phenotype correlation. Overall, the phenotypic variability demonstrated by this cohort, prompts a high index of clinical suspicion even in cases with two of the three cardinal features (ACC, GDD, bilateral congenital cataract). Furthermore, considering the severe GDD, special attention should be drawn to vision (as later onset visual impairment is possible), hearing, as well as delineating the source of the hearing impairment, and continued neurology and endocrine follow-up. Cardiomyopathy is a common finding in Vici syndrome, and as seen in our cohort, both dilated and hypertrophic cardiomyopathy have been observed (Cullup et al., 2013; Byrne et al., 2016a; Abidi et al., 2020), with an age of onset ranging from antenatal (Touraine et al., 2017) to several years.

To conclude, our findings contribute to the current knowledge regarding the molecular basis and phenotypic features of Vici syndrome, including its prenatal presentation. Additionally, the deep learning-based facial gestalt adds to the clinician’s diagnostic toolbox and may aid in facilitating identification of additional individuals affected with this unique and rare disorder.

## Data Availability

The datasets for this article are not publicly available due to concerns regarding participant/patient anonymity. Requests to access the datasets should be directed to the corresponding author.
