# Supplementary material for: Vici syndrome in Israel: Clinical and molecular insights
Source: Front Genet. 2022 Sep 20;13:991721. doi: 10.3389/fgene.2022.991721 (PMC9531146; doi:10.3389/fgene.2022.991721)
Supplement: Supplementary file 1 [file Table1.docx]

**Supplementary Table 1 -** Clinical and molecular characteristics of individuals harboring disease causing bi-allelic variants in *EPG5*

| Patient | A1* | A2* | B1 | B2 | C1** | D1 | E1* | E2* | E3* | E4* | E5 |
| --- | --- | --- | --- | --- | --- | --- | --- | --- | --- | --- | --- |
| Family | A | | B | | C | D | E | | | | |
| Additional affected family members | +  First cousins | | +  One child, 3 TOP: 2 males, one female, all with ACC | | - | - | +  Siblings (E1, E2,E3), Cousins (E4, E5) | | | | |
| Family history of cancer | - | | +  Mother, maternal grandfather: recurrent melanoma,  Mother, maternal grandfather and maternal uncles: GI polyps;  Maternal grandmother: papillary thyroid cancer; maternal uncle: CRC;  Extended family with CRC | | - | +  Mother- gastric cancer,  Maternal grandfather diagnosed with lung and colon cancer at the age of ~75 years | - | | | | |
| ***Molecular diagnosis*** | | | | | | | | | | | |
| Variant(s) in *EPG5*  NM_020964.3 | Homozygous  c.3447G>A;  p.Trp1149* | Homozygous  c.3447G>A;  p.Trp1149* | No DNA available (assumed to be compound heterozygote as Case B2) | Compound heterozygous  c.1007A>G;  p .Gln336Arg  (paternally inherited);  c.2554-5A>G  (maternally inherited) | Homozygous  c.1007A>G;  p .Gln336Arg | Homozygous  c.1461del  p.Ala488LeufsTer32 | Homozygous  c.5993C>G,p.Ser1998* | Homozygous  c.5993C>G,p.Ser1998* | Homozygous  c.5993C>G,p.Ser1998* | Homozygous  c.5993C>G,p.Ser1998* | Homozygous  c.5993C>G,p.Ser1998* |
| Diagnostic test | Targeted gene sequencing | Targeted gene sequencing | Father- founder mutation sequencing; mother- gene panel | Sanger sequencing | WES | WES | Sanger sequencing | Sanger sequencing | Sanger sequencing | Sanger sequencing | Sanger sequencing |
| ***Demographic data*** | | | | | | | | | | | |
| Sex | F | M | M | F | F | F | M | M | F | F | M |
| Ethnicity | Arab-Muslim | Arab-Muslim | Ashkenazi-Jewish | Ashkenazi-Jewish | Ashkenazi-Jewish | Ashkenazi-Jewish | Arab-Muslim | Arab-Muslim | Arab-Muslim | Arab-Muslim | Arab-Muslim |
| Parental consanguinity | + | + | - | - | - | *+ | + | + | + | + | + |
| Age at last follow up | Deceased at 2.5 years | Deceased at 2.8 years | Deceased at ֿ  7 years | TOP | 3.5 years | 18 months | Deceased at 4.5 years | Deceased at 8 months | Deceased at 17 months | Deceased at 5 months | 15 months old |
| ***Growth*** | | | | | | | | | | | |
| Weight | -6 SD | -6 SD | NA | NA | -3SD | -3.5 SD | NA | NA | NA | NA | -3.2 SD |
| Head circumference (cm) | -4 SD | -2 SD | Normal at birth, progressive microcephaly | NA | NA | -2.2 SD | NA | NA | NA | NA | NA |
| Feeding difficulties requiring PEG insertion | + | + | + | NA | +**** | +**** | + | + | + | + | + |
| ***Neurological manifestations*** | | | | | | | | | | | |
| Gross motor developmental delay (no milestones) | + | + | + | NA | + | + | + | + | + | + | + |
| Fine motor developmental delay (severe) | + | + | + | NA | + | + | + | + | + | + | + |
| Speech and communication delay (severe) | + | + | + | NA | + | + | + | + | + | + | + |
| Hypotonia | + | + | + | NA | + | + | + | + | + | + | + |
| Myopathy | -  Normal EMG | +  Muscle biopsy: numerous autophagosomes | +  Muscle biopsy suspected mitochondrial myopathy | NA | NA | NA | +  Muscle biopsy: suspected mitochondrial abnormalities | +  Muscle biopsy  moderate mitochondrial abnormalities, | NA | NA | NA |
| Seizures | +  and opisthotonus  (Tx: Phenobarbital) | +  (Tx: Phenobarbital) | + | NA | NA | +  (Tx: Topiramate & Levetiracetam) | NA | NA | NA | NA | +  (Tx: Levetiracetam) |
| Brain MRI findings | ACC, pontine cerebellar hypoplasia, ventriculomegaly | ACC, large citerna magna and delayed myelination | ACC | Partial ACC | ACC, dysgenesis of brainstem and cerebellum, dilated third ventricle, colpocephaly of cerebral ventricles | ACC, enlarged lateral ventricles, brain atrophy, agenesis of septum pellucidum | ACC | ACC | ACC | ACC | ACC |
| ***Dysmorphic facial features*** | | | | | | | | | | | |
| Dysmorphic facial features | NA | High palate , Polydactyly in both feet | High palate | NA | High palate anteverted nostrils, long philtrum, unusual hair, cutis marmorta, hip contractures | High palate low-set posteriorly rotated ears, frontal bossing, retrognathia, arachnodactyly | NA | NA | NA | NA | NA |
| ***Pigmentation*** | | | | | | | | | | | |
| Hair and skin hypopigmentation | + | + | - | NA | + | + | + | + | + | + | + |
| Multiple hemangiomas | NA | NA | - | NA | + | - | NA | NA | NA | NA | - |
| ***Ophthalmologic and ENT manifestations*** | | | | | | | | | | | |
| Cataract | + | - | + | + (per autopsy) | + | + | + | + | + | + | + |
| Additional ophthalmological findings | - | - | Nystagmus, pigmentary retinopathy | - | bilateral optic nerve hypoplasia | - | - | - | - | - | bilateral optic nerve atrophy, chorioretinitis |
| Hearing loss | + | + | NA | NA | NA | - | NA | NA | NA | + | + |
| ***Cardiac involvement*** | | | | | | | | | | | |
| Cardiomyopathy | +  Concentric hypertrophy of left ventricle with normal ventricular function | +  Dilated cardiomyopathy with some hypertrophy | - | NA | +  Mild dilated cardiomyopathy | +  Severe hypertrophic cardiomyopathy | +  Hypertrophic cardiomyopathy | +  Hypertrophic cardiomyopathy | +  Hypertrophic cardiomyopathy | +  Hypertrophic cardiomyopathy | +  Dilated cardiomyopathy |
| Medical treatment | Furosemide, Spironolactone, Captopril | Furosemide, Spironolactone, | NA | NA | Enalapril (later halted due to worsening of cough) | Atenolol, Disopyramide, Amlodipine | NA | NA | NA | NA | Captopril |
| ***Immune deficiency*** | | | | | | | | | | | |
| Recurrent upper respiratory tract infections and pneumonia | + | + | + | NA | +  CT: bronchiectasis | + | + | + | + | + | + |
| Other | - | - | Recurrent urinary tract infections, Varicella | NA | Low IgA, IgG immunoglobulins, orbital cellulitis, milk allergy (FPIES) | Relative hypergammaglobulinemia (IgA, IgG), T and B cell repertoire within normal limits | - | - | Chronic diarrhea | Chronic diarrhea | Neutropenia |
| ***Laboratory abnormalities*** | | | | | | | | | | | |
| Elevated CPK | + | - | + | NA | + | - | + | + | + | + | + |
| Elevated liver enzymes | + | + | + | NA | + | + | + | + | + | + | + |
| ***Additional features*** | | | | | | | | | | | |
| Additional features | +  Susp bilateral DDH | +  Bilateral toe polydactyly | +  Right cryptorchidism | NA | +  Contractures, central hypoventilation milk allergy (FPIES) | +  Hashimoto’s Disease, Hypertension | - | - | - | - | - |

AHI, apnea hypopnea index; CC, corpus callosum; CPK, creatine phosphokinase; CRC, colorectal cancer; CT, computed tomography; DCM, dilated cardiomyopathy; EMG, electromyography; ENT, ear nose and throat; FPIES, food protein-induced enterocolitis syndrome; GI, gastrointestinal; Hypertrophic cardiomyopathy; LE, left eye; MRI, magnetic resonance imaging; NA, not available; NCV, nerve conduction velocity; PEG, percutaneous endoscopic gastrostomy; SD, standard deviation; TOP, termination of pregnancy; Tx, treatment; US, ultrasound; UTI, urinary tract infection; WES, whole exome sequencing.

* This patient was mentioned in a previous publication: Byrne S, et al. EPG5-related Vici syndrome: a paradigm of neurodevelopmental disorders with defective autophagy. Brain 2016;139(Pt 3):765-81.

** This patient was briefly mentioned in our recent publication: Pode-Shakked B, et al. A single center experience with publicly funded clinical exome sequencing for neurodevelopmental disorders or multiple congenital anomalies. Sci Rep 2021;11(1):19099.

***Parents are third degree cousins.
