# Supplementary figures and images for: Vici syndrome in Israel: Clinical and molecular insights
Source: Front Genet. 2022 Sep 20;13:991721. doi: 10.3389/fgene.2022.991721 (PMC9531146; doi:10.3389/fgene.2022.991721)

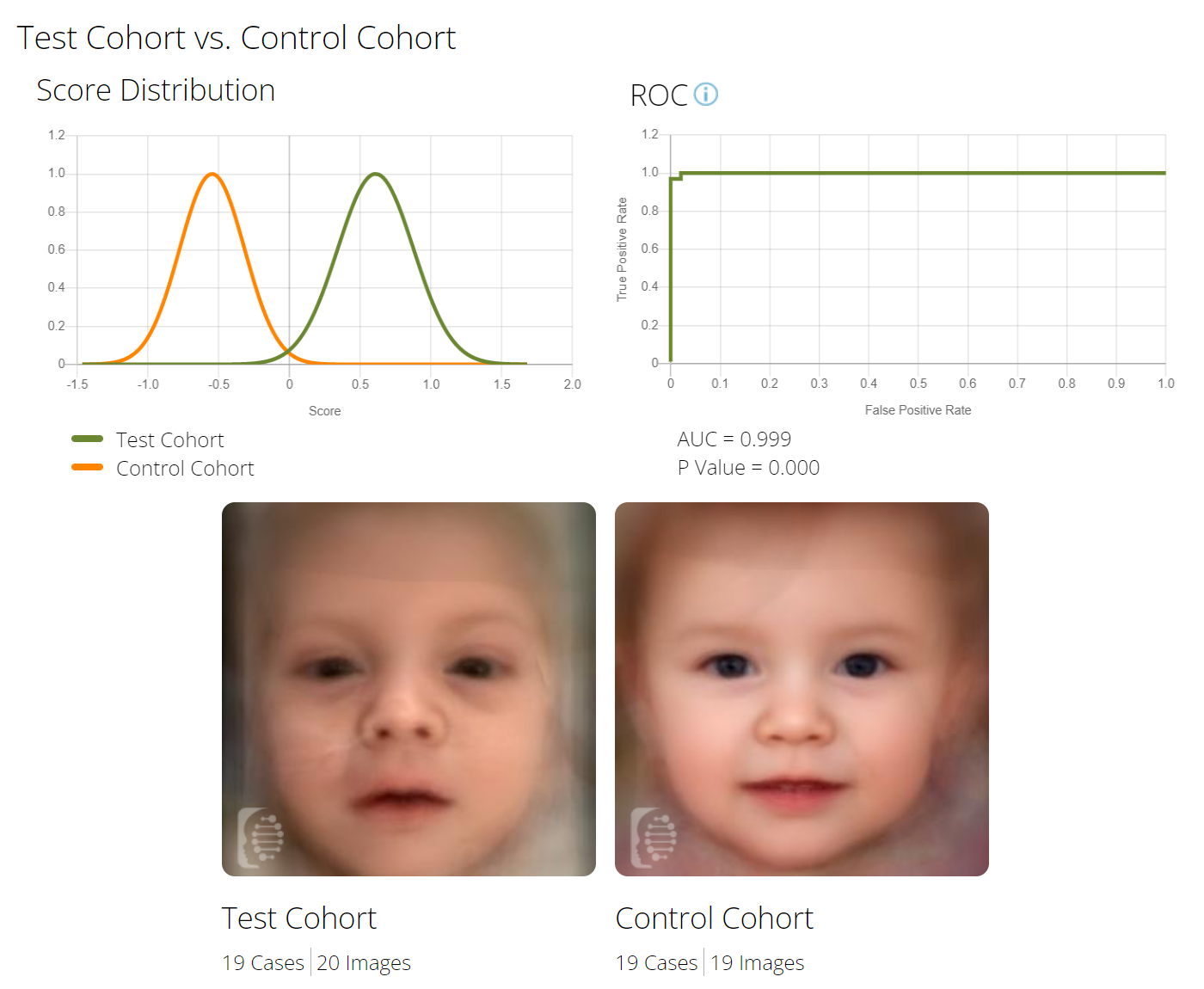

Supplement: Supplementary file 2 [file Image1.PNG]
