# Supplementary material for: Vici syndrome in Israel: Clinical and molecular insights
Source: Front Genet. 2022 Sep 20;13:991721. doi: 10.3389/fgene.2022.991721 (PMC9531146; doi:10.3389/fgene.2022.991721)
Supplement: Supplementary file 3 [file DataSheet1.docx]

**Supplemental Material for:**

**Vici syndrome in Israel: Clinical and molecular insights**

## Detailed clinical descriptions

**Family A** presented with two first degree cousins (Patients A1 and A2) of Muslim Arab descent. Both patients (a female (A1) and a male (A2)) presented postnatally with severe GDD, seizures and hearing loss of unknown etiology. Physical features included dysmorphic features and hypopigmentation of hair and skin. Brain imaging revealed agenesis of corpus callosum, along with additional findings. Progressive growth retardation requiring gastrostomy insertion developed soon after birth, without improvement of growth parameters. Dilated cardiomyopathy with reduced ventricular function was diagnosed and treated. The patients also experienced recurrent infections.

Patient A1 died at the age of 2.5 years, and patient A2 died at 2 years and 8 months following respiratory infections. Of note, these two patients were previously reported by Byrne *et al.* (2016).

**Family B**, of Ashkenazi-Jewish descent and no known consanguinity, presented with two cases, one detected prenatally following which the pregnancy was terminated.

The first male patient (B1) presented postnatally with severe developmental delay, marked hypotonia and seizures. Additional features included congenital cataract, agenesis of the corpus callosum and recurrent infections. Patient B1 died at the age of 7 years.

Case B2, a female fetus diagnosed prenatally with ultrasonography (US) findings of partial agenesis of the corpus callosum. Following a genetic molecular diagnosis, termination of pregnancy was performed. Fetal autopsy revealed bilateral cataracts.

Of note, two additional pregnancies of the couple were terminated following prenatal findings of partial agenesis of the corpus callosum. Unfortunately, further data regarding their prenatal features or molecular diagnosis were unavailable, although family history is highly suggestive of recurring Vici syndrome in these 3 fetuses.

Family history was notable for several cases of malignancies, including recurrent melanoma in the mother and maternal grandfather, colorectal cancer in a sibling of the maternal grandfather, papillary thyroid carcinoma in the maternal grandmother and multiple persons with colorectal polyps (mother, maternal uncles, maternal grandparents).

**Family C**, of Ashkenazi-Jewish (Poland) descent with no known consanguinity, presented with a female infant (Patient C1). Prenatal findings included agenesis of the corpus callosum, dysgenesis of the brainstem and cerebellum, dilated third ventricle, and colpocephaly of cerebral ventricles. The parents opted to proceed with the pregnancy following genetic counselling. Postnatally, the patient presented with severe developmental delay and marked hypotonia. Physical features included hypopigmented hair and skin, with dysmorphic features (Figure 2Ac). Progressive growth retardation and feeding difficulties developed soon after birth, and milk allergy (FPIES) was diagnosed. The patient underwent percutaneous endoscopic gastrostomy (PEG) insertion and Nissen fundoplication, with resolution of allergic symptoms towards one year of age. Visual involvement was first noted at one year of age, with bilateral optic nerve hypoplasia and cataract. Mild dilated cardiomyopathy developed after one year of age. Recurrent respiratory infections, and central hypoventilation were noted (AHI of 14.6, 9.8 central index and 20.2/hr index of desaturations), and treated with oxygen supplementation. The patient is currently 3.5 years old.

The proband of **Family D** (Patient D1), born to consanguineous parents (3^rd^ degree cousins) of Ashkenazi-Jewish descent (Germany), presented during early infancy with severe developmental delay and marked hypotonia. Physical features included gold-colored hair and skin hypopigmentation, along with dysmorphic facial features (Figure 2Ad) and bilateral cataracts. Progressive growth retardation and feeding difficulties developed soon after birth and the patient underwent PEG insertion and Nissen fundoplication. Brain MRI was notable for agenesis of corpus callosum along with additional features. Hypertrophic cardiomyopathy was diagnosed at the age of six weeks and recurrent respiratory infections ensued. Patient D1 is currently 1.5 years old.

Her family history is notable for maternal invasive liver cancer (age 29) and a maternal grandfather diagnosed with lung and colon cancer at the age of ~75 years.

**Family E**, of Arab-Muslim descent, consists of 5 patients born to highly consanguineous parents. Patients E1, E2 and E3 are siblings, while Patients E4 and E5 are their cousins. Patients E1-E4 were previously reported by Byrne et al., 2016. Prenatal findings in the fifth case (Patient E5) included agenesis of corpus callosum, and given the family history, prenatal genetic counselling was conducted with a suspicion for Vici Syndrome, and the family opted to continue with the pregnancy. All affected family members presented with severe developmental delay and marked hypotonia. Physical features included hypopigmented hair and skin, with dysmorphic features (Figure 2Aa-f). Progressive growth retardation and feeding difficulties developed soon after birth, and patients underwent percutaneous endoscopic gastrostomy (PEG) insertion. All cases presented with bilateral cataract and cardiac involvement. Of the three siblings, patient E1 died at the age of 4.5 years, patient E2 died at the age of 8 months and patient E3 died at the age of 17 months following respiratory infections. Patient E4 died at the age of 5 months, and patient E5 is currently 15 months old.
